# Supplementary material for: Assessing real-time Zika risk in the United States
Source: BMC Infect Dis. 2017 May 4;17:284. doi: 10.1186/s12879-017-2394-9 (PMC5415743; doi:10.1186/s12879-017-2394-9)
Supplement: Supplementary file 1 — Supplemental Information: A framework for assessing real-time Zika risk in the United States. (PDF 10870 kb) [file 12879_2017_2394_MOESM1_ESM.pdf]

Supplement: A framework for assessing real-time Zika risk in the United States

Lauren A Castro [1*], Spencer J Fox[1*@], Xi Chen[2], Kai Liu[3], Steve Bellan[4], Nedialko B Dimitrov[2], Alison P Galvani[5,6], Lauren Ancel Meyers[1,7],

1 Section of Integrative Biology, The University of Texas at Austin, Austin, TX, USA
2 Graduate Program in Operations Research Industrial Engineering, The University of Texas at Austin, Austin, TX, USA
3 Institute for Cellular and Molecular Biology, The University of Texas at Austin, Austin,TX, USA
4 Center for Computational Biology and Bioinformatics, The University of Texas at Austin, Austin, TX, USA
5 Center for Infectious Disease Modeling and Analysis, Yale School of Public Health, New Haven, CT, USA
6 Department of Ecology and Evolution, Yale University, New Haven, CT, USA
7 The Santa Fe Institute, Santa Fe, NM, USA

*contributed equally to this manuscript
@ corresponding author: spncrfx@gmail.com

Methodological Overview

This manuscript introduces a framework for interpreting Zika Virus (ZIKV) surveillance data in the face of uncertainty regarding importation, transmission, and reporting rates. In this supplement, we provide additional methodological details (Sections 1-3), Texas county risk assessments (Section 3), and parameter sensitive analyses (Section 4). Sections 1-3 detail each of the three major steps of our methodology: (1) estimating county-specific ZIKV transmission and importation risks, (2) simulating ZIKV outbreaks using a probabilistic branching process ZIKV transmission model, and (3) analyzing simulations to estimate risk and provide analytic tools for interpreting surveillance data (Fig 1).

1 Texas County Risk Assessment

1.1 County Importation Rate Estimation

We assumed that ZIKV outbreaks originate with the arrival (importation) of an infectious individual and built a model to estimate county-level ZIKV importation rates across Texas. The model consists of two components, estimated separately and then multiplied to obtain county importation rates: (1) a statewide ZIKV importation rate and (2) county-specific probabilities of receiving the next Texas importation. The main text describes our estimation of statewide ZIKV importation rates; here, we detail our methods for estimating county-level probabilities. County-to-county variation

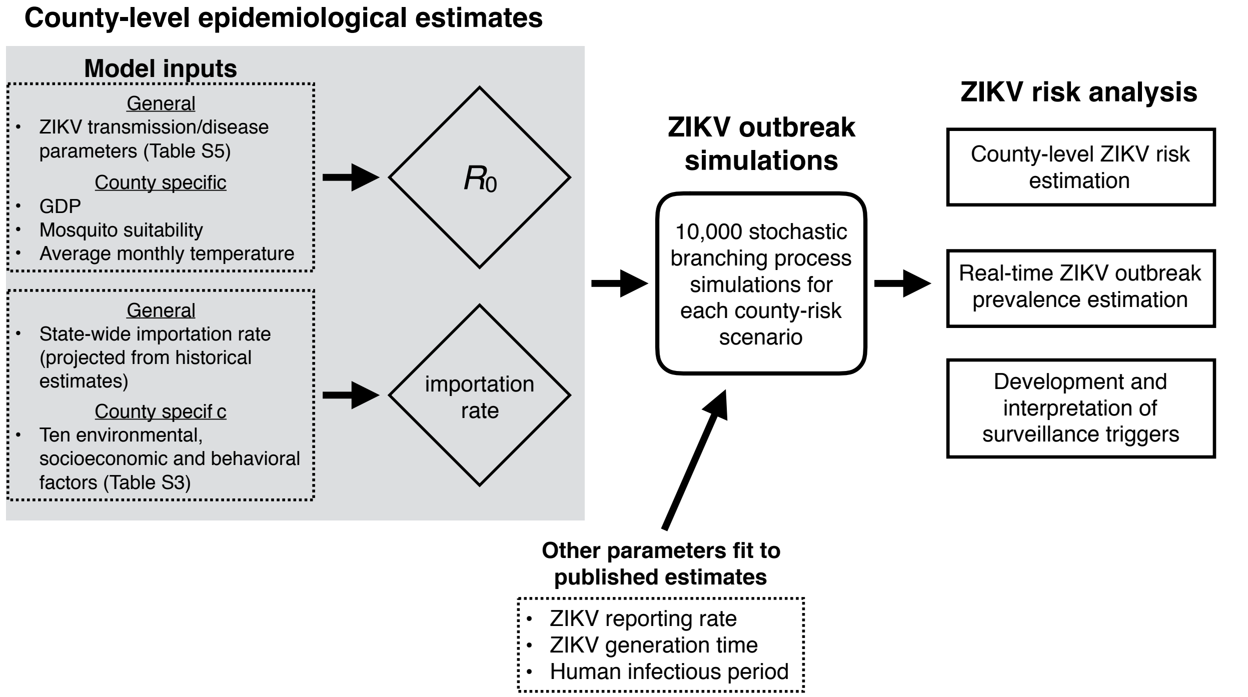

Figure 1: **ZIKV Risk Assessment Framework.** The method consists of three steps. First, we use data-derived models to estimate county-level ZIKV introduction rates and ZIKV transmission rates. Each estimate is based on a combination of general and county-specific factors. Second, for every county-risk combination, we simulate 10,000 ZIKV outbreaks using a stochastic branching process ZIKV transmission model parameterized by the county-level importation and transmission rate estimates along with several other recently published disease progression estimates. The simulations track the numbers of autochthonous and imported cases (unreported and reported) and, based on the total size and maximum daily prevalence, classifies each outbreak as self-limiting or epidemic. Third, we analyze the simulations to determine (1) robust relationships between the number of reported cases in a county and the current and future ZIKV threat and (2) surveillance triggers (number of reported cases) indicative of imminent epidemic expansion.

in importation rates may be influenced by a number of different environmental, socioeconomic, and behavioral factors. To build a predictive model consisting of a tractable number of informative predictors, we fit a maximum entropy model consisting of dozens of possible predictors to historical arbovirus importation data, and then pruned the model through two rounds of variable selection to avoid model overfitting.

1.1.1 Historical Arbovirus Data

We analyzed ten years (2002-2012) of Dengue (DENV) importation data (number of importations in a county) provided by the Texas Department of State Health Services (DSHS), two years of CHIKV importation data scraped from online DSHS reports, and the 30 recent ZIKV importations into Texas counties from January 2, 2016 to April 1, 2016 [1, 2]. Given the geographic and biological overlap between ZIKV, DENV, and CHIKV, we use historical DENV and CHIKV importation data to supplement ZIKV importations. We believe this use of DENV and CHIKV data is reasonable, given that the importation model fit only to DENV and CHIKV data predicts the county ZIKV importation distribution well [3]. Importation rates should be primarily governed by international travel into Texas from affected regions and fairly insensitive to differences in modes of transmission (e.g., different vector species and sexual transmission).

Currently, DENV, CHIKV, ZIKV importation patterns differ most noticeably along the Texas-Mexico border. Endemic DENV transmission and sporadic CHIKV outbreaks in Mexico historically have spilled over into neighboring Texas counties. We included DENV and CHIKV importation data in the model fitting so as to consider potential future importations pressure from Mexico, as ZIKV cases continue to increase in Mexico.

1.1.2 Sociological Predictor Data

The socioeconomic and demographic predictors for county-level ZIKV importation risk include population size, employment status, population below poverty, modes of commuting to work, and health insurance coverage, which we obtained from the 2009-2013 American Community Survey 5-year estimates `https://www.census.gov/acs/www/data/data-tables-and-tools/data-profiles/2013/`. In lieu of data regarding travel to ZIKV affected regions (which are not available), we also considered the size of tourism industry for each county by collecting economic impact of travel data from Dean Runyan Associates report `http://www.deanrunyan.com/doc_library/TXImp.pdf` . Our full model included 72 factors across four categories (Table 4).

1.1.3 Maximum Entropy Model

Let $X = \{x_1, x_2, ..., x_{254}\}$ represent the 254 counties of Texas. Suppose a case of ZIKV is introduced into Texas and let p_i be the probability that it occurs in county x_i. The vector of p_i sums to one over all counties. We wish to estimate this unknown probability distribution using the historical import data. The relative probabilities $p_1, p_2, ...p_n$ can be constrained with known mean, variance, or other moments of some known $f_j(X)$ for each county. The functions $f_j(X)$ are functions of socioeconomic, environmental, and travel variables in our case (Table 4). Mathematically, we want to:

$$\max_{p_i} \quad \Big(-\sum_{i=1}^{254} p_i \log p_i\Big) \tag{1a}$$

$$\text{s.t.} \qquad \sum_{i=1}^{254} p_i f_j(x_i) = E(f_j(X)) \quad \forall j \tag{1b}$$

$$\sum_{i=1}^{254} p_i = 1 \tag{1c}$$

$$p_i \geq 0 \qquad \forall i \tag{1d}$$

When we use Shannon's measure of entropy as the objective (1a), the constraints (1d) are automatically satisfied. The right-hand-side of (1b), $E(f_j(X))$, is estimated by the weighted arithmetic mean of $f_j(x_1), f_j(x_2), ..., f_j(x_{254})$ based on the 254 counties of Texas [4].

1.1.4 Representative Variable Selection

Before solving the maximum entropy model, we first removed duplicate variables or positively correlated variables —variables that bring the essentially identical information to the model. Selecting the representative variables was done with a variation of the facility location problem [5]. The goal was to select k variables that adequately represent the entire variable set. The k selected factors would each represent themselves and the remaining $72 - k$ variables would be represented by exactly one variable from the k selected variables (Eq 2b). The $\ell{-}\infty$ norm of the difference between two unit-norm variables, denoted by f_i, f_j in Table 1, was used to measure the distance between pairs of variables. This distance measure was derived from the maximum difference in expectations that the two variables can produce, under any probability distribution. The facility location model allowed us to select the k most representative variables (Eq (2c)). The objective function (Eq 2a) for selecting representative variables was to minimize the distance between the k representative variables and all the variables in the entire variable set. The results of the representative variable selection procedure are showed in Fig 2a.

$$\min_{x_{ij}, y_j} \quad \sum_{i=1}^{n}\sum_{j=1}^{n} d_{ij} x_{ij} \tag{2a}$$

$$\text{s.t.} \quad \sum_{j=1}^{n} x_{ij} = 1 \qquad \forall i \tag{2b}$$

$$\sum_{j=1}^{n} y_j = k \tag{2c}$$

$$x_{ij} \leq y_j \quad \forall i, j \tag{2d}$$

$$x_{ij} \in \{0,1\} \quad \forall i, j \tag{2e}$$

$$y_j \in \{0,1\} \qquad \forall j \tag{2f}$$

1.1.5 Predictive Variable Selection

To further streamline the importation model, we considered several different methods for identifying the most predictive among the selected variables, including hypothesis testing to choose between

Symbol	Definition
f_j	72 variables represented by vectors $f_j, j = 1, 2, ...72$
d_{ij}	distance between two variables, measured as $d_{ij} = \| \frac{f_i}{\|f_i\|_2} - \frac{f_j}{\|f_j\|_2} \|_\infty$
x_{ij}	$x_{ij} = 1$ if vector i is represented by vector j; $x_{ij} = 0$, otherwise;
y_j	$y_j = 1$, if vector j is selected as representative vector; $y_j = 0$, otherwise;

Table 1: **Representative variable selection. We first applied this method to reduce our county-level ZIKV importation model from 72 to 20 predictors, and then applied predictive variable selection to reduce it further to 10 predictors.**

nested models [6]. Ultimately, we applied a backward selection approach, outlined in Table 2. In each iteration, the variable that contributed the least to model performance was dropped, until all the variables were eliminated. Specifically, we evaluated model performance through cross validation on out-of-sample data to avoid overfitting the model. For each iteration, 12 years of DENV and CHIKV importation cases were divided into two subsets: a 9 year training data set (for fitting the model) and a 3 year testing data set (for gauging model performance). We ran each set of variables on 6 randomly selected partitions of the 12 years of available data. From the 6 runs, we calculated the average of the out-of-sample log-likelihood of the model and eliminated the variable that gave the largest mean out-of-sample log-likelihood. Drops in model performance was negligible until fewer than 10 variables were included. Results of predictive variable selection procedure are showed in Fig 2b.

Algorithm	Backward Selection
1	**function** BACKWARD SELECTION (N)
2	Set $V = N$
3	**While** $\|V\| > 1$ **do**
4	Set $e = argmax_{e \in V} C(S(V - e))$
5	Set $V = V - \{e\}$
6	Record V and $C(S(V - e))$
N	The complete set of representative variables
C	Return the out-of-sample log-likelihood, averaged over of seven randomly sampled cross validation folds
S	Fit a maximum entropy model given a set of variables f_j

Table 2: **Predictive variable selection algorithm used to select the 10 most informative variables from among the 20 representative variables selected for the Texas county ZIKV importation model.**

Variables ordered by importance
Total Amount of County Direct Spending on Traveling ($K)
Percentage Population holding Graduate or professional degree
Total Amount of Visitor Tax Receipts(Local) ($K)
County Male Population
Population Commuting to Work with Other Means
Max Temperature of Warmest Month
Percentage Population below Poverty Level
Precipitation of Wettest Quarter
Population without Health Insurance
Population holding Graduate or professional degree

Table 3: **Import risk model variables**. These 10 variables were selected from 72 variables using a combination of representative variables selection and predictive backwards selection. The importance of each variable (from top to bottom) is determined by order of exclusion in backwards selection, with the most important variables remaining in the model the longest.

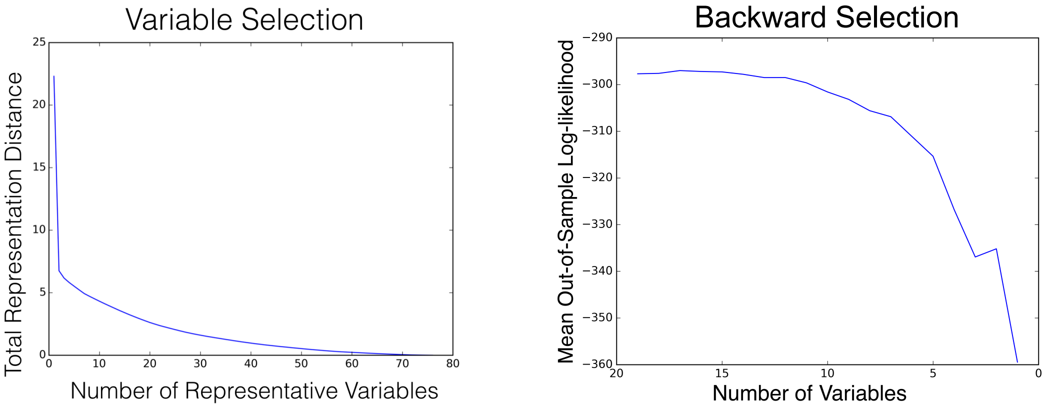

(a) Representative Variables Selection

(b) Predictive Variable Selection

Figure 2: **Import Risk Variable Selection Criteria.** Methods for down selecting predictor variables from 72 to 10. (A) Representative Variable Selection: Representation distance – a measure of how much information is dropped, with higher distance dropping more information – versus number of representative variables. (B) Backwards Selection: Mean out–of-sample log-likelihood versus number of variables used in the model.

Environmental	Socio-economic	Demographic, Travel and Vector Suitability
Annual Mean Temperature	Employed Population	Male Population
Annual Precipitation	Unemployed Population	Female Population
Slope	Employed Population in Percentage	Male Population in Percentage
Population Count	Unemployed Population in Percentage	Female Population in Percentage
Isothermality	Population below Poverty Level in Percentage	Total Amount of Visitor Tax Receipts(Local)
Precipitation of Driest Month	Families below Poverty Level in Percentage	Total Amount of Visitor Tax Receipts(State)
Elevation	Population with Health Insurance	Total Amount of County Direct Spending on Traveling
Maximum Green Vegetation Cover	Percentage with Health Insurance	Total Amount of Visitor Spending
Temperature Seasonality	Population without Health Insurance	Total Amount of Travel Earnings
Precipitation Seasonality	Percentage without Health Insurance	Travel Related Number of Jobs
Min Temperature of Coldest Month	Population Walk to Work in Percentage	Average MGV (percentage per km)
Precipitation of Driest Quarter	Percentage Population Commuting to Work with Taxi	Total Approximate MGV Cover (km)
Max Temperature of Warmest Month	Mean Population Travel Time to Work(Minutes)	
Precipitation of Wettest Quarter	Population Walk to Work	
Temperature Annual Range	Population Commuting to Work with Taxi	
Precipitation of Warmest Quarter	Percentage Commuting to Work with Public Transportation	
Mean Temperature of Wettest Quarter	Commuting to Work with Public Transportation	
Precipitation of Coldest Quarter	Commuting to Work with Car, Truck or Van (Carpooled)	
Mean Temperature of Driest Quarter	Commuting to Work with Car, Truck or Van(Alone)	
Mean Temperature of Warmest Quarter	Percentage Population Commuting to Work with Car, Truck or Van(Carpooled)	
Mean Temperature of Coldest Quarter	Percentage Population Commuting to Work with Car, Truck or Van(Alone)	
Mean Diurnal Range	Population Commuting to Work with Other Means	
Precipitation of Wettest Month	Percentage Commuting to Work with Other Means	
Aspect	Education Attainment below 9th grade	
Artificial Surface Cover(Percentage)	Percentage Population Education Attainment below 9th grade	
Total Artificial Surface Cover (km)	Population with Education Attainment between 9th and 12th grade	
	Percentage Population Education Attainment between 9th and 12th grade	
	Population with High School Graduation	
	Percentage Population with High School Graduation	
	Population go to College without diploma	
	Percentage Population go to College without diploma	
	Population with Associates degree	
	Percentage of Population with Associates degree	
	Population with Bachelor's degree	
	Percentage Population with Bachelor's degree	
	Population with Graduate or professional degree	
	Percentage of Population with Graduate or professional degree	

Table 4: **All 72 environmental, socioeconomic, and behavioral variables evaluated for Texas county ZIKV importation model are at a county level from the 2009-2013 American Community Survey 5-year estimates** https://www.census.gov/acs/www/data/data-tables-and-tools/data-profiles/2013/**, Dean Runyan Associates report** http://www.deanrunyan.com/doc_library/TXImp.pdf **and WorldClim Database** [7]

1.2 County R_0 Estimation

1.2.1 Data

***Aedes aegypti* abundance** We assumed that mosquito abundance in a given county is distributed as a Poisson random variable, with expectation λ = -ln(1-county occurrence probability) [8]. For each county, we first estimated occurrence probability by averaging the $5km^2$ occurrence probabilities given in [9] contained within the county, and then applied the equation above to derive an average county abundance.

In this analysis we focus exclusively on *Ae. aegypti* as the primary vector of ZIKV transmission. However, experimental infections of *Ae. albopictus* in Florida found that this species is also capable of transmitting ZIKV, but has a lower probability of infection than *Ae. aegypti.* In Texas, the distribution of *Ae. albopictus* largely overlaps with that of *Ae. aegypti* but extends further westward [9]. These western areas are expected to receive negligible ZIKV importations, whereas the areas of vector overlap include high ZIKV importation zones such as the southern border and the Houston metropolitan area. Thus, by excluding *Ae. albopictus* from our analysis, we may be underestimating relative risk in some of the highest risk areas, but this will depend on the vectorial competence of *Ae. albopictus.* We also do not consider the common species *Culex quinquefasciatus*, as there is laboratory evidence that it is not competent to transmit ZIKV in humans [10], although recent statistical work predicts that it may be competent [11].

Average temperatures For each county we obtained average minimum and maximum temperatures for each month for each county (C°) from 2000 to 2010 using the CDC Wonder interface [12]. We averaged the minimum and maximum temperatures to obtain estimates for the average monthly temperatures.

Gross Domestic Product We obtained county GDP from 2010 as part of the G-Econ research project (http://gecon.yale.edu/) [13].

1.2.2 R_0 calculation

Perkins *et. al.* generated functional distributions via 1000 Monte Carlo samples from the underlying uncertainty in the parameter distributions (available at https://github.com/TAlexPerkins/Zika_nmicrobiol_2016). We combined these functionals with county average temperature and GDP estimates, to obtain a county R_0 distribution. Given the high uncertainty in the underlying parameter estimates, we chose to analyze relative risk by scaling the median R_0 estimate of each county from 0 (lowest risk)-1 (highest risk). For the purpose of demonstration, we calculated a county's sustained transmission rate using a product of the relative risk and a plausible maximum R_0. In the analysis we chose this R_0 to be 1.5.

2 ZIKV Outbreak Simulations

2.1 Simulating Outbreaks

We model ZIKV outbreaks using a stochastic Markov branching process model (Fig 4). To transmit ZIKV, a mosquito must bite an infected human, get infected with the virus, bite a susceptible human, and the human must then get infected with the virus. Rather than explicitly model the full transmission cycle, we aggregate the two-part cycle of ZIKV transmission (mosquito-to-human and human-to-mosquito) into a single exposed class, and do not explicitly model mosquitoes. For the

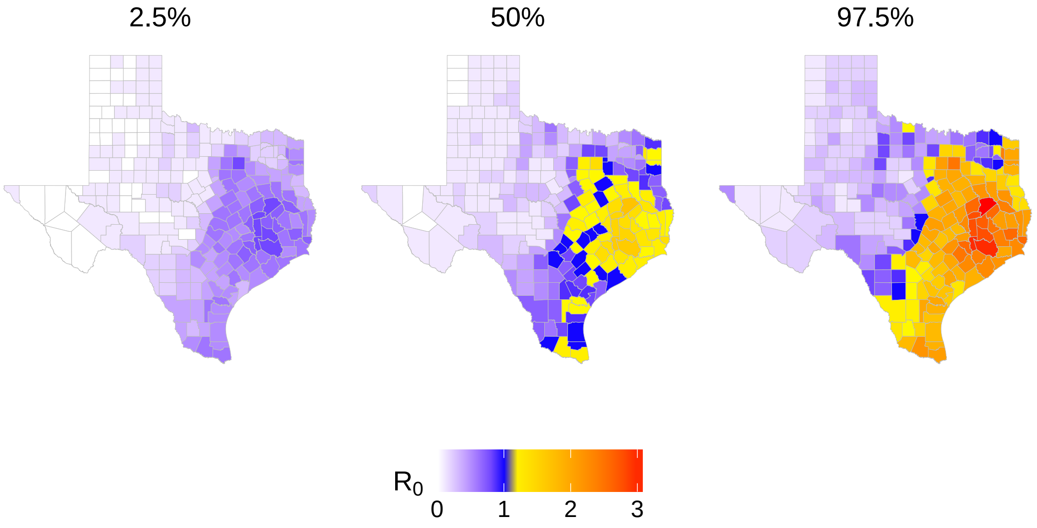

Figure 3: **The 95% CI of R_0 Distributions for August.** From left to right, the 2.5%, 50% and 97.5% quantile R_0 values for August. The range of absolute values spans 0.02-6.90. Given the considerable uncertainty in socioeconomic and environmental drivers of ZIKV, we analyzed relative rather than absolute transmission risks.

purposes of this study, we need only ensure that the model produces a realistic human-to-human generation time of ZIKV transmission.

Our simulations begin with a single ZIKV imported case, and we simulate the Susceptible-Exposed-Infectious-Recovered (SEIR) transmission process that follows. The temporal evolution of the compartments are governed by daily probabilities for infected individuals transitioning between E, I and R states, new ZIKV introductions and transmissions, and reporting of current infectious cases (Table 5). We assume that infectious cases cause a Poisson distributed number of secondary cases per day (via human to mosquito to human transmission), and that low reporting rates correspond to the percentage ($\sim 20\%$) of symptomatic ZIKV infections [14]. Although reporting has been as low as 10% in historical ZIKV outbreaks, we focus primarily on 20% reporting for the majority of results. We make the simplifying assumption that asymptomatic cases transmit ZIKV at the same rate as symptomatic cases, which can be modified if future evidence suggests otherwise. We give the model equations below for both introduced cases (Eqs 3) and autochthonous cases (Eqs 4).

For each scenario, consisting of a particular importation rate, transmission rate, and reporting rate, we ran 10,000 stochastic simulations. Each simulation began with a single infectious unreported importation and terminated when there were no individuals in either the *Exposed* or *Infectious* classes or the cumulative number of autochthonous infections reached 2,000. We classified simulations as either epidemics or self-limiting outbreaks; epidemics are those reaching 2,000 cumulative autochthonous infections with a maximum daily prevalence of at least 50 (Fig 6). We define daily prevalence as the number of current unreported and reported autochthonous infections.

2.2 Model Equations: Introduced ZIKV Cases

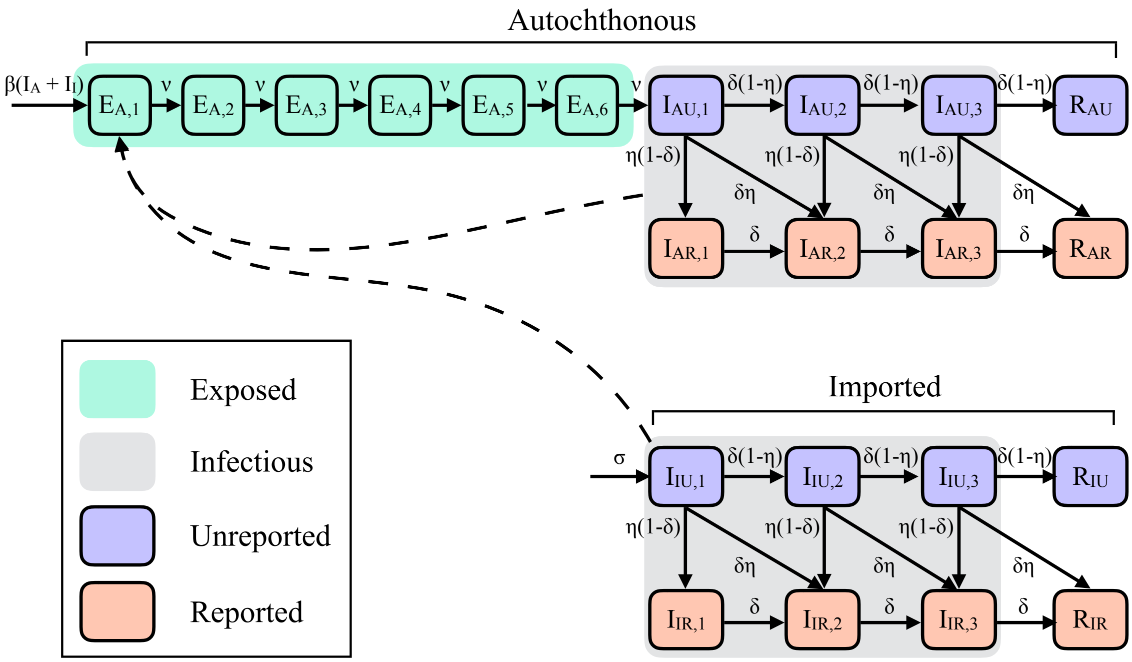

Figure 4: **Diagram of ZIKV outbreak model.** The model tracks disease progression, transmission, and reporting of both imported and autochthonous ZIKV cases. Individuals progress through compartments via a daily Markovian process, according to the solid arrows in the diagram. The *Exposed* and *Infectious* periods consist of several (boxcar) compartments to simulate realistic outbreak timing. Unreported infected individuals have a daily probability of being reported. Imported cases are assumed to arrive daily according to a Poisson distribution (with mean σ) at the beginning of their infectious period, and otherwise follow the same infectious process as autochthonous cases. Autochthonous transmission occurs at rate $\beta(I_A + I_I)$, where I_A and I_I are the total number of infectious autochthonous and imported cases, respectively (dashed arrows).

Unreported compartments:

$$I_{IU,1}(t) = I_{IU,1}(t-1) + Pois\,(x=1, p=\sigma) - (\delta + \eta - \delta\eta)I_{IU,1}(t-1) \tag{3a}$$

For $i \in 2, \ldots, n$ infectious compartments:

$$I_{IU,i}(t) = I_{IU,i}(t-1) + \delta(1-\eta)I_{IU,i-1}(t-1) - (\delta + \eta - \delta\eta)I_{IU,i}(t-1) \tag{3b}$$

$$R_{IU}(t) = R_{IU}(t-1) + \delta(1-\eta)I_{IU,n}(t-1) \tag{3c}$$

Reported compartments:

$$I_{IR,1}(t) = I_{IR,1}(t-1) + \eta(1-\delta)I_{IU,1}(t-1) - (\delta)I_{IR,1}(t-1) \tag{3d}$$

For $i \in 2, \ldots, n$ infectious compartments:

$$\begin{aligned} I_{IR,i}(t) = I_{IR,i}(t-1) + (\delta)I_{IR,i-1}(t-1) + (\delta\eta)I_{IU,i-1}(t-1) + \eta(1-\delta)I_{IU,i}(t-1) \\ - (\delta)I_{IR,i}(t-1) \end{aligned} \tag{3e}$$

$$R_{IR}(t) = R_{IR}(t-1) + (\delta)I_{IR,n}(t-1) + (\delta\eta)I_{IU,n}(t-1) \tag{3f}$$

2.3 Model Equations: Autochthonous ZIKV Cases

Exposed compartments:

$$E_{A,1}(t) = E_{A,1}(t-1) + \sum \left(Pois \left(x = \sum_{j \in \{IU,IR,AU,AR\}} \sum_{k=1}^{n} I_{j,k}, \;\; p = \beta \right) \right) - (\nu) E_{A,1}(t-1) \tag{4a}$$

For $i \in 2, \ldots, e$ exposed compartments:

$$E_{A,i}(t) = E_{A,i}(t-1) + (\nu) E_{A,i-1}(t-1) - (\nu) E_{A,i}(t-1) \tag{4b}$$

Unreported compartments:

$$I_{AU,1}(t) = I_{AU,1}(t-1) + (\nu) E_{A,e}(t-1) - (\delta + \eta - \delta\eta) I_{AU,1}(t-1) \tag{4c}$$

For $i \in 2, \ldots, n$ infectious compartments:

$$I_{AU,i}(t) = I_{AU,i}(t-1) + \delta(1-\eta) I_{AU,i-1}(t-1) - (\delta + \eta - \delta\eta) I_{AU,i}(t-1) \tag{4d}$$

$$R_{AU}(t) = R_{AU}(t-1) + \delta(1-\eta) I_{AU,n}(t-1) \tag{4e}$$

Reported compartments:

$$I_{AR,1}(t) = I_{AR,1}(t-1) + \eta(1-\delta) I_{AU,1}(t-1) - (\delta) I_{AR,1}(t-1) \tag{4f}$$

For $i \in 2, \ldots, n$ infectious compartments:

$$I_{AR,i}(t) = I_{AR,i}(t-1) + (\delta) I_{AR,i-1}(t-1) + (\delta\eta) I_{AU,i-1}(t-1) + \eta(1-\delta) I_{AU,i}(t-1) - (\delta) I_{AR,i}(t-1) \tag{4g}$$

$$R_{AR}(t) = R_{AR}(t-1) + (\delta) I_{AR,n}(t-1) + (\delta\eta) I_{AU,n}(t-1) \tag{4h}$$

With $Pois\,(x,p)$ indicating x random draws from a Poisson distribution with $\lambda = p$, subscripts of I and A, respectively, indicating introduced and autochthonous cases, subscripts of R and U, respectively, indicating reported and unreported cases, and parameter values defined as described in Table 5.

2.4 Fitting the Generation Time

To capture the correct outbreak timing, we fit the generation time of our SEIR model to estimates for the ZIKV exposure and infectious periods in humans. The generation time measures the average duration from initial symptom onset to the subsequent exposure of a secondary case, and is estimated to range from 10 to 23 days for ZIKV [16]. In our model, the generation time corresponds to the sum of the exposure period and 1/2 the infectious period. We therefore fit the infectious period in our model to human ZIKV estimates for duration of viral shedding, and then fit the exposure period so that the sum of the two classes match the estimated ZIKV serial interval.

Parameter	Description	Values Investigated (or median 95%)	Source
Exposed compartments (e)	Number of exposed compartments	6	Fit (See 2.4)
Incubation Rate (ν)	Daily probability of progressing from one exposed compartment to the next	0.584	[15, 16]
Infectious compartments (n)	Number of infectious compartments	3	Fit (See 2.4)
Recovery Rate (δ)	Daily probability of progressing from one infectious compartment to the next	0.3041	[15, 16]
Reproduction Number (R_0)	The expected total number of secondary infections from one infectious individual in a fully susceptible population	$0 - 3.1$	County R_0 estimates
Daily Reporting Rate (η)	The daily probability of an infectious individual being reported	Daily: $0.011 - 0.0224$ Overall: $10 - 20\%$	[14]
Daily Importation Rate (σ)	The expected number of infectious ZIKV importations per day	$0.0 - 1.21$	County importation rate estimates
Generation Time	The average length of time between consecutive exposures $GT = \frac{e}{\nu} + (\frac{1}{2})\frac{n}{\delta}$	15 (9.5-23.5) days	[16]

Table 5: **Stochastic ZIKV outbreak model parameters**. We hold the disease progression parameters constant across all scenarios, estimate R_0 and importation rate for each individual county, and vary the reporting rate to investigate its impact on the uncertainty of ZIKV risk assessments.

According to our modeling framework: with one infectious compartment, the distribution of waiting times in the compartment would follow a geometric distribution, with the most common waiting time equal to one day regardless of the transition rate. As this is a biologically unrealistic waiting time distribution, we use Boxcar implementations to yield a more realistic distribution [17]. In such a framework one splits a compartment into multiple separate compartments (boxes), has individuals transition through these compartments, and alters the transition rate for each compartment so the average waiting time spent in all compartments equals that of the original desired average. For example, if a 10 day infectious period were desired, one could model the infectious period as 1 compartment with a daily transition rate of 1/10, or 5 compartments with a daily transition rate of 5/10. The number of infectious individuals is either the number of individuals in the single compartment, or the total number of individuals in all five boxes. Both scenarios would have an average waiting time of 10 days to move through the infectious period, but the 5 boxes would necessitate individuals being infectious for at least 5 days giving a more realistic waiting time distribution that follows a negative binomial distribution (sum of multiple independent geometric distributions).

First, we solved for transition rates and compartments of a Boxcar Model infectious period that yielded an infectious period with 3 compartments and mean duration of 9.88 days and 95% CI of (3-22) [15]. Then, we fit the exposure period so that the combined duration of the infectious and exposure periods matched the empirical ZIKV generation time range [16], yielding 6 compartments and a mean exposure period of 10.4 days (95% CI 6-17) and finally a mean generation time of 15.3 days (95% CI 9.5-23.5). Given that the exposure period includes human and mosquito incubation periods and mosquito biting rates, this range is consistent with the estimated 5.9 day human ZIKV incubation period [15]

3 Risk Assessment and Surveillance Trigger Analysis

Although ZIKV surveillance data will ultimately be used for many planning and response purposes, here we focus on just one: assessing the potential for epidemic expansion. This is intended as a demonstration and test of the approach, which can be similarly applied to plan and improve other surveillance activities.

We classify simulations as epidemics if the reaches at least 2,000 autochthonous cases (Our simulation length - Fig 5)and, at least once, surpass a daily autochthonous prevalence of 50. The second criteria was systematically designed to distinguish (1) simulations with high transmission rates from (2) simulations with low transmission rates but high importation rates (Fig 6). The daily prevalence threshold of 50 ensures that the vast majority of outbreaks and epidemics are classified correctly. Occasionally, outbreaks with $R_0 \leq 1$ and a high importation rate grow sufficiently large to be classified as epidemics, even though they technically are not. Since they would warrant substantial a public health response [18], we let the classification stand. As discussed in the main text, such misclassifications arise only under exceedingly high importations rates and do not qualitatively influence our results.

To find the epidemic risk in a county upon seeing, x, reported cases, we first find all trials in our 10,000 simulations that encounter x reported cases, and then find what proportion of those simulations become an epidemic. For example, if 1,000 of a county's simulated outbreaks have 2 reported cases, but only 50 of those simulations become epidemics, than the epidemic risk upon seeing 2 cases in that county would be 5%. This framework allows us to assess 1) the likelihood of a county experiencing x reported cases and 2) the probability of sustained transmission upon a second reported cases (assuming no subsequent intervention).

We only analyze triggers for counties where at least 1% of simulations reach the trigger value (number of reported cases), to avoid accidentally inflating the risk of counties that have only very few simulations reaching the trigger value. This method also does not distinguish between counties with 500 epidemics out of 1,000 *triggered* simulations from those with 5,000 of 10,000. Thus, we report the probability of a *triggered* outbreak separately. Consider a county with an $R_0 = 1.1$ and another county with a much higher R_0. In the second county, outbreaks are much more likely to progress into epidemics. However, both counties should interpret a cluster of reported cases as strong indication of epidemic expansion, regardless of the prior probability that such a cluster would occur.

We evaluate epidemic risk across Texas counties following two reported autochthonous cases, in line with recent CDC's guidelines [18]. As demonstrated in the main text, our framework can also be applied to design surveillance triggers, based on local epidemic risks and reporting rates. We show our full Texas risk assessment under a worse case elevated importation scenario in Fig **??**.

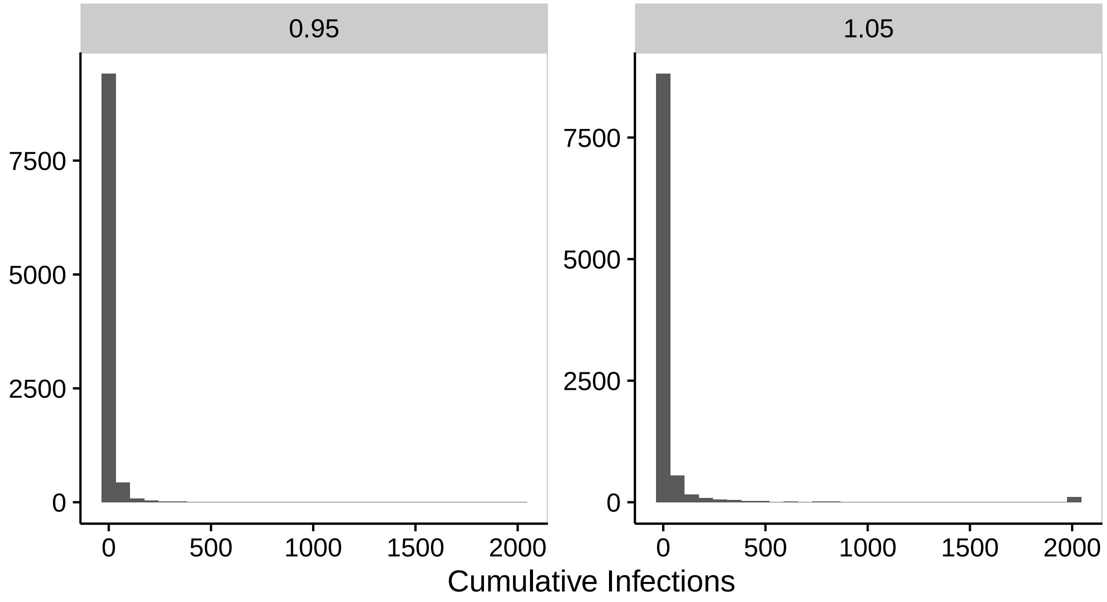

Figure 5: **Determining outbreak simulation length.** If outbreak simulations are too short, self-limiting outbreaks may reach the maximum number of infections due to stochasticity. We chose to run our simulations to 2,000 cumulative infections as it conservatively differentiated the large outbreaks of simulations with R_0 just below 1 (0.95) from the epidemics of those with R_0 just above 1 (1.05). We therefore chose to run our simulations until a maximum number of 2,000 infections.

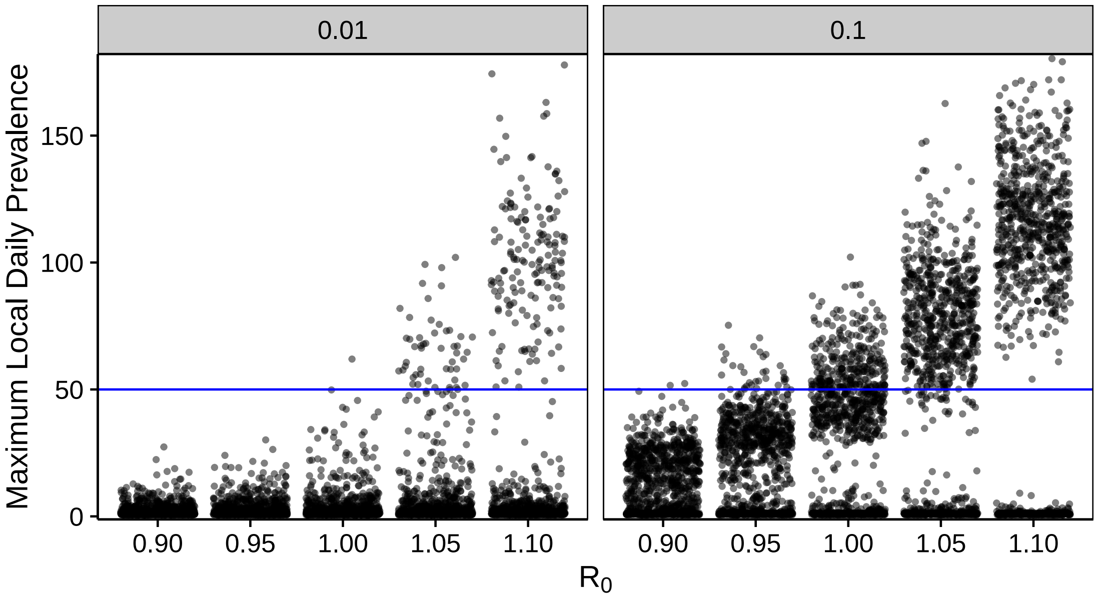

Figure 6: **Selecting daily prevalence threshold for distinguishing self-limiting outbreaks from epidemics.** Across a range of R_0 values, we plot the maximum daily total autochthonous infectious individuals for 1,000 of our 10,000 trials (black dots). The blue line indicates the threshold (50) selected to differentiate epidemics with $R_0 > 1$ from outbreaks with $R_0 \leq 1$. At a low importation rate (0.01), the majority of simulations with $R_0 \leq 1$ are self-limiting and rarely progress into large sustained outbreaks. As R_0 increases, a greater proportion of simulations exceed the threshold. As the importation rate increases (panels from left to right) the separation between self-limiting outbreaks and epidemics becomes more pronounced.

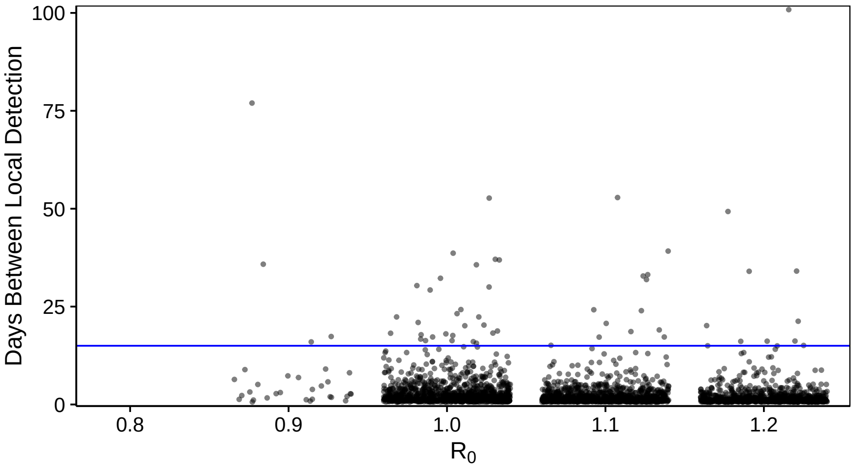

Figure 7: **Time between detection of locally transmitted cases during epidemics.** Across a range of R_0 values with an importation rate 0.1 cases/day, we plot the time between detection events of autochthonous cases for simulations out of the 10,000 trials in which epidemics occurred (black dots). The blue line indicates a two-week threshold as recommended by the CDC for follow-up of local transmission. Even under a high importation rate of 0.1 cases/day, epidemics do not occur when $R_0 = 0.8$, and rarely occur when $R_0 = 0.9$. As R_0 increases, a greater proportion of simulations have fewer days in between detection events as the number of infections rapidly increase.

3.1 Texas risk assessment

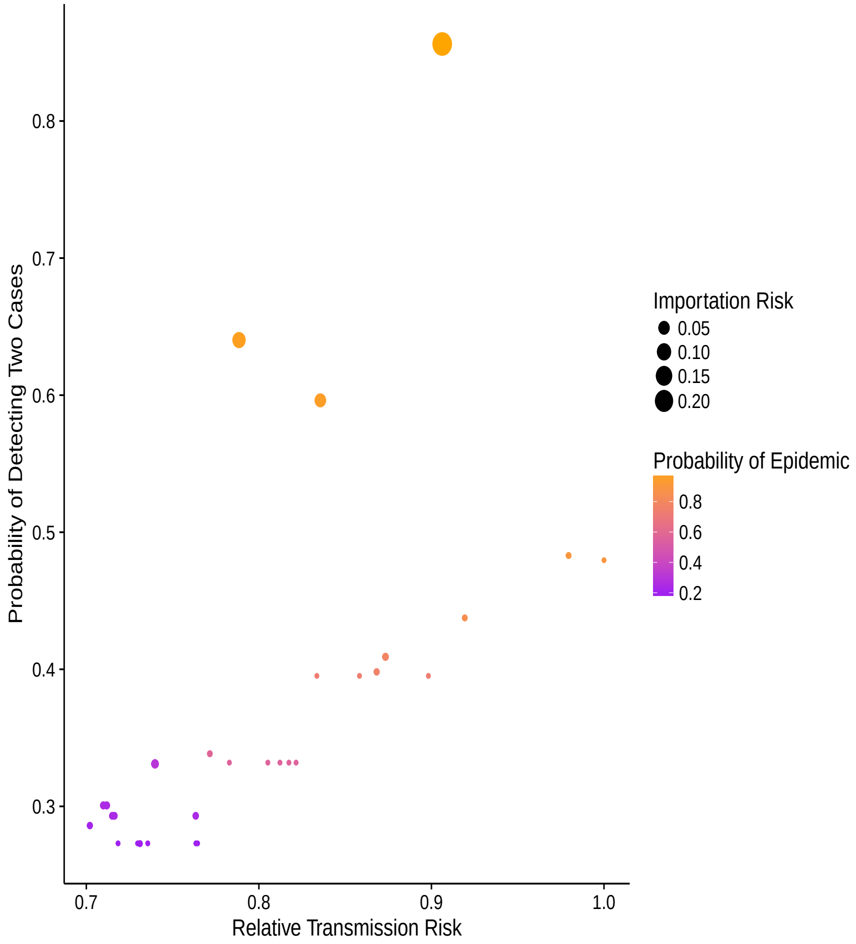

Figure 8: **Interactions between county importation rate, sustained transmission risk, and epidemic probability** The estimated sustained transmission risk is plotted against the probability of detecting two cases in the county. It is clear that there is a baseline monotonically increasing risk for detecting two cases as sustained transmission risk increases. Abnormally large importation risks (point size), alter this relationship to drastically increase the probability of detecting two cases within a county. The probability of an epidemic upon seeing two cases (point colors) is best predicted by the sustained transmission risk and not the importation risk unless the importation risk is high.

4 Seasonal Analysis

The extrinsic incubation period of ZIKV in *Ae. aegypti* and the mortality rate of *Ae. aegypti* are hypothesized to be temperature dependent, as reported for other *Flaviviridae* viruses [19, 20, 21], though they have not yet been directly estimated for Texas *Ae. aegypti*. Here, we consider seasonal changes in these transmission drivers and its impact on the distribution of relative risk across Texas. Specifically, we calculate monthly R_0 estimates throughout the summer and fall of 2016,

based on historical monthly average temperatures (from 1980-2010) for each county [12] (Fig 9). We normalized R_0 values across all months to show the relative transmission risk, using the overall maximum R_0 as the highest risk county and month. Transmission risk is expected to be stable throughout the summer, and into September. In October, however, the number of higher risk counties is expected to decrease, reamining concentrated in the southern (warmer) counties. Based on historical temperature differences between months, by November the relative transmission risk is expected to be low across Texas.

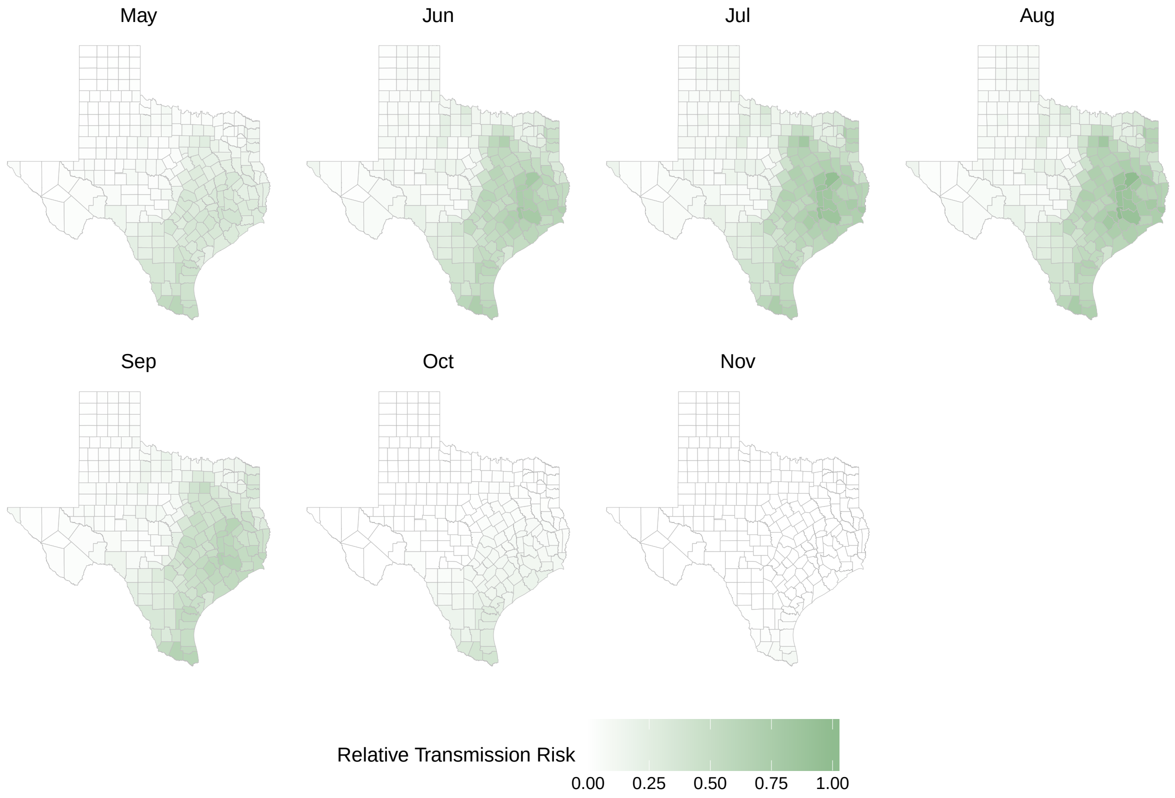

Figure 9: **Monthly R_0 estimates based on seasonal changes in the temperature-dependent extrinsic incubation period of ZIKV in *Ae. aegypti* and the mosquito mortality rate of *Ae. aegypti*.**

References

[1] Texas Department of State Health and Human Services. Arbovirus Activity in Texas 2013 Surveillance Report. Texas Department of State Health Services; Infectious Disease Control Unit Zoonosis Control Branch; 2013.

[2] Texas Department of State Health and Human Services. Arbovirus Activity in Texas 2014 Surveillance Report. Texas Department of State Health Services-Infectious Disease Control Unit Zoonosis Control Branch; 2014.

[3] Texas Arbovirus Risk. The University of Texas:Scholar Works; 2015. Available from: http://repositories.lib.utexas.edu/handle/2152/31934.

[4] Kapur JN, Kesavan HK. Entropy optimization principles with applications. Academic Pr; 1992.

[5] Wolsey LA. Integer programming. vol. 42. Wiley New York; 1998.

[6] Halvorsen R, Mazzoni S, Bryn A, Bakkestuen V. Opportunities for improved distribution modelling practice via a strict maximum likelihood interpretation of MaxEnt. Ecography. 2015;38(2):172–183.

[7] Hijmans RJ, Cameron SE, Parra JL, Jones PG, Jarvis A, et al. Very high resolution interpolated climate surfaces for global land areas. Int J Climatol. 2005;25(15):1965–1978.

[8] Perkins TA, Siraj AS, Ruktanonchai CW, Kraemer MU, Tatem AJ. Model-based projections of Zika virus infections in childbearing women in the Americas. Nature Microbiology. 2016;1:16126.

[9] Kraemer MUG, Sinka ME, Duda KA, Mylne A, Shearer FM, Barker CM, et al. The global distribution of the arbovirus vectors Aedes aegypti and Ae. albopictus. eLife [Internet]. 2015 Jun [cited 2016 Apr 19];4:e08347. Available from: http://elifesciences.org/content/4/e08347v3.

[10] Fernandes RS, Campos SS, Ferreira-de Brito A, de Miranda RM, da Silva KAB, de Castro MG, et al. Culex quinquefasciatus from Rio de Janeiro is not competent to transmit the local Zika virus. PLoS Negl Trop Dis. 2016;10(9):e0004993.

[11] Evans MV, Dallas TA, Han BA, Murdock CC, Drake JM. Data-driven identification of potential Zika virus vectors. eLife. 2017 feb;6:e22053. Available from: https://dx.doi.org/10.7554/eLife.22053.

[12] North America Land Data Assimilation System (NLDAS) Daily Air Temperatures and Heat Index, years 1979-2011. CDC WONDER Online Database; released 2012.

[13] Nordhaus WD. Geography and macroeconomics: New data and new findings. Proceedings of the National Academy of Sciences of the United States of America. 2006;103(10):3510–3517.

[14] Duffy MR, Chen TH, Hancock WT, Powers AM, Kool JL, Lanciotti RS, et al. Zika virus outbreak on Yap Island, Federated States of Micronesia. New Eng J Med [Internet]. 2009 Jun [cited 2016 Apr 4];360(24):2536–2543. Available from: http://www.nejm.org/doi/abs/10.1056/NEJMoa0805715.

[15] Lessler J, Ott CT, Carcelen AC, Konikoff JM, Williamson J, Bi Q, et al. Times to Key Events in the Course of Zika Infection and their Implications for Surveillance: A Systematic Review and Pooled Analysis. bioRxiv [Internet]. 2016 Mar [cited 2016 Apr 26];Available from: http://biorxiv.org/content/early/2016/03/02/041913.abstract.

[16] Majumder MS, Cohn E, Fish D, Brownstein JS. Estimating a feasible serial interval range for Zika fever. Bulletin of the World Health Organization [Internet]. 2016 [cited 2016 May 10];Available from: http://www.who.int/bulletin/online_first/16-171009.pdf.

[17] Lloyd AL. Realistic Distributions of Infectious Periods in Epidemic Models: Changing Patterns of Persistence and Dynamics. Theoretical Population Biology. 2001;60(1):59–71. Available from: http://www.sciencedirect.com/science/article/pii/S0040580901915254.

[18] Centers for Disease Control and Prevention. Draft Interim CDC Zika Response Plan (CONUS and Hawaii): Initial Response to Zika Virus. 2016;Atlanta, Georgia.

[19] Chan M, Johansson MA. The incubation periods of dengue viruses. PloS one. 2012;7(11):e50972.

[20] Scott TW, Amerasinghe PH, Morrison AC, Lorenz LH, Clark GG, Strickman D, et al. Longitudinal studies of Aedes aegypti (Diptera: Culicidae) in Thailand and Puerto Rico: blood feeding frequency. Journal of medical entomology. 2000;37(1):89–101.

[21] Black WC, Bennett KE, Gorrochótegui-Escalante N, Barillas-Mury CV, Fernández-Salas I, de Lourdes Muñoz M, et al. Flavivirus susceptibility in Aedes aegypti. Archives of medical research. 2002;33(4):379–388.
